# Ciprofloxacin triggered glutamate production by *Corynebacterium glutamicum*

**DOI:** 10.1186/s12866-016-0857-6

**Published:** 2016-10-07

**Authors:** Dorit Lubitz, Volker F. Wendisch

**Affiliations:** Genetics of Prokaryotes, Faculty of Biology and Center for Biotechnology, Bielefeld University, Universitätsstraße 25, 33615 Bielefeld, Germany

**Keywords:** *Corynebacterium glutamicum*, Ciprofloxacin, DNA gyrase, Glutamate, Ornithine, Putrescine, Arginine, Lysine, 2-oxoglutarate, Overflow metabolism

## Abstract

**Background:**

*Corynebacterium glutamicum* is a well-studied bacterium which naturally overproduces glutamate when induced by an elicitor. Glutamate production is accompanied by decreased 2-oxoglutatate dehydrogenase activity. Elicitors of glutamate production by *C. glutamicum* analyzed to molecular detail target the cell envelope.

**Results:**

Ciprofloxacin, an inhibitor of bacterial DNA gyrase and topoisomerase IV, was shown to inhibit growth of *C. glutamicum* wild type with concomitant excretion of glutamate. Enzyme assays showed that 2-oxoglutarate dehydrogenase activity was decreased due to ciprofloxacin addition. Transcriptome analysis revealed that this inhibitor of DNA gyrase increased RNA levels of genes involved in DNA synthesis, repair and modification. Glutamate production triggered by ciprofloxacin led to glutamate titers of up to 37 ± 1 mM and a substrate specific glutamate yield of 0.13 g/g. Even in the absence of the putative glutamate exporter gene *yggB*, ciprofloxacin effectively triggered glutamate production. When *C. glutamicum* wild type was cultivated under nitrogen-limiting conditions, 2-oxoglutarate rather than glutamate was produced as consequence of exposure to ciprofloxacin. Recombinant *C. glutamicum* strains overproducing lysine, arginine, ornithine, and putrescine, respectively, secreted glutamate instead of the desired amino acid when exposed to ciprofloxacin.

**Conclusions:**

Ciprofloxacin induced DNA synthesis and repair genes, reduced 2-oxoglutarate dehydrogenase activity and elicited glutamate production by *C. glutamicum*. Production of 2-oxoglutarate could be triggered by ciprofloxacin under nitrogen-limiting conditions.

**Electronic supplementary material:**

The online version of this article (doi:10.1186/s12866-016-0857-6) contains supplementary material, which is available to authorized users.

## Background

Glutamic acid and its salts are used as flavor enhancers since decades, due to its “meaty” taste, designated as “umami” [1]. The annual global production of glutamic acid and its salts amounts to about three million tons per year and is still increasing [2]. *Corynebacterium glutamicum* was discovered because it naturally excretes high amounts of glutamate under certain conditions [3, 4]. Due to this ability, *C. glutamicum* and its close relatives are used for the industrial production of glutamate [2, 5]. This rod shaped, Gram-positive bacterium is biotin auxotrophic and secretes glutamate, for instance when biotin is limiting [4]. Biotin has to be supplemented to the growth media to maintain the function of the two enzymes pyruvate carboxylase (EC 6.4.1.1) and acetyl-CoA carboxylase (EC 6.4.1.2) [6, 7]. The second catalyzes the first committed step in fatty acid synthesis [7]. Thus, biotin limitation may be closely connected to changes in the membrane composition. It has also been shown, that glutamate production, induced by biotin limitation, is always accompanied with membrane alteration. However, membrane alterations alone are not a sufficient prerequisite for the production of glutamate [8]. Other membrane destabilizers like detergents (surfactants) or fatty acids like Polyoxyethylen(20)-sorbitan-monopalmitate (Tween-40) and cell wall affecting compounds are used to elicit glutamate production in *C. glutamicum* [9–15]. For example, Penicillin G which inhibits the transpeptidase activity and, thus, cross-linking of cell wall peptidoglycan is a commonly known elicitor of glutamate production by *C. glutamicum* [16, 17]. Another antibiotic affecting cell wall synthesis is ethambutol which inhibits the arabinosyltransferase, an enzyme involved in the polymerization of cell wall arabinogalactan [18–20].

Although the described elicitors for glutamate production affect the cell envelope of *C. glutamicum*, it is widely accepted that membrane alteration alone is not sufficient for glutamate production and the ‘leak model’ is obsolete [8, 21]. On the one hand, under several glutamate overproducing conditions the metabolic flux is changed, because 2-oxoglutarate dehydrogenase complex (ODHC) activity is reduced [22]. This is contributed to the inhibition of ODHC via OdhI [23, 24]. On the other hand, it is evident that glutamate is not only diffusing through the membrane passively, but involves active export [21, 25]. Recent results affirm that active glutamate export is due to the putative mechanosensitive channel protein YggB [26, 27]. Triggering glutamate overproduction by *C. glutamicum* is a complex phenomenon, but a growth limitation per se (e.g., due to phosphate limitation, [28]) does not lead to glutamate overproduction. It has also been established that triggering export alone is not sufficient for glutamate overproduction [20, 21, 29–31]. However, all known triggers of glutamate overproduction lead to reduced ODHC activity [22, 32–34]. The underlying regulatory mechanism is not transcriptional regulation, but inhibition of ODHC on the enzyme activity level by OdhI, a specific inhibitory protein [24, 35, 36].

Since the beginning of investigation of glutamate production by *C. glutamicum* it is known that agents targeting the DNA synthesis can elicit the production of glutamate [37]. Nevertheless, none of these inhibitors of DNA replication were analyzed in *C. glutamicum* regarding their mode of action in glutamate synthesis. Ciprofloxacin, a member of the fluoroquinolone antibiotics, inhibits DNA gyrase and topoisomerase IV of Gram-negative as well as of Gram-positive bacteria [38, 39]. Therefore, it causes the stagnation of the cell division due to its DNA replication inhibiting function. Here, we report that ciprofloxacin does not only arrest growth of *C. glutamicum*, but also triggers glutamate production.

## Results

### Effects of ciprofloxacin on colony formation

Typically production of a desired metabolite, for instance an amino acid, occurs at the expense of biomass formation. Therefore, arresting growth while maintaining substrate utilization should lead to higher product yields. While sub-lethal concentrations of cell wall active antibiotics such as penicillin G are known to trigger glutamate production by *C. glutamicum*, the effect of ciprofloxacin, an inhibitor of DNA gyrase and topoisomerase IV in Gram-positive bacteria and, thus, of DNA synthesis [38, 39], on *C. glutamicum* has not been tested. It is believed that bacterial cells exposed to ciprofloxacin are non-dividing, but living and metabolically active [38].

**Fig. 1:**
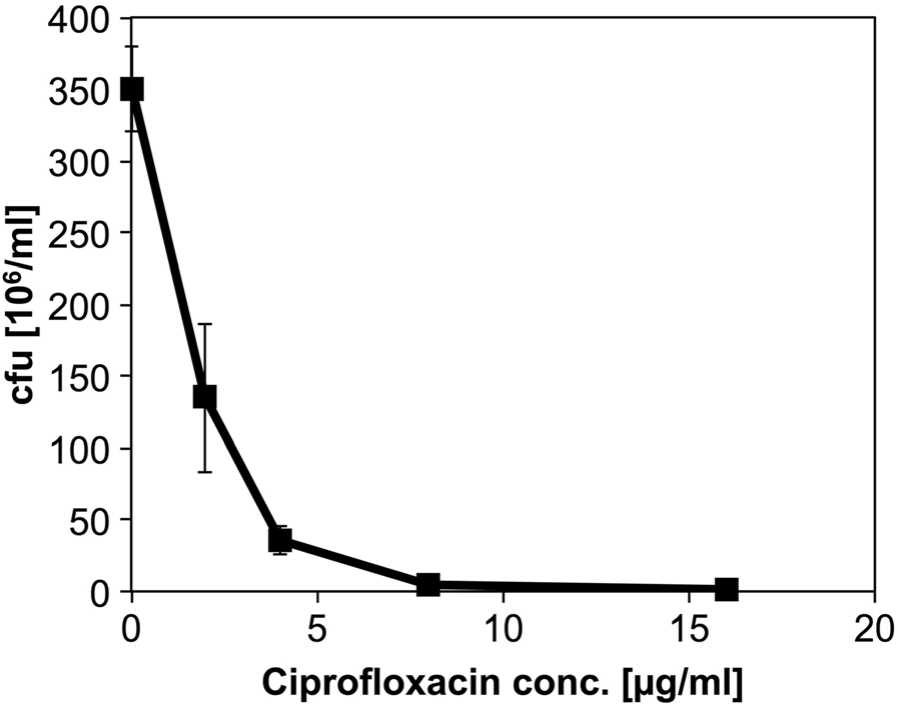
Colony formation of *C. glutamicum* wild type in the presence of different ciprofloxacin concentrations. The cells were cultured in CGXII (4 % (w/v) glucose) to an OD_600_ of 15 and ciprofloxacin was added. After five hours of ciprofloxacin exposure, cells were diluted in 0.9 % NaCl to an OD_600_ of 1 and further diluted. Colony forming units (cfu) were determined. Experiments were performed in biological duplicates and colony number determined for two technical replicates

To determine how ciprofloxacin affects *C. glutamicum,* cells growing exponentially on glucose minimal medium were exposed to ciprofloxacin for five hours before the colony forming units (cfu) were determined. Growth was arrested already at very low ciprofloxacin concentrations (IC_50_ = 1.3 μg/ml), for example at a concentration of 4 μg/ml, the ability to form colonies was already reduced by 90 % (Fig. 1). Colony formation at high ciprofloxacin concentrations was heterogeneous whereas untreated cells formed uniform colonies (see Additional file 1: Figure S1).

### Transcriptional effects due to addition of ciprofloxacin

To determine the transcriptional response of *C. glutamicum* to sub-inhibitory ciprofloxacin concentrations, a microarray experiment was performed for comparison of differential gene expression due to the exposure to ciprofloxacin. Therefore, the *C. glutamicum* WT was cultured in CGXII and 1 % (w/v) glucose to an OD600 of about 5 before no or 4 μg∙ml^-1^ ciprofloxacin were added for one hour. This relatively low concentration was used, because the growth inhibitory effects are less severe than at higher concentrations. The differential expression of ciprofloxacin treated cells was compared to untreated cells (Table 1). Mostly, genes important for DNA synthesis, repair or modification such as *recA* (codes for DNA recombinase A), *cglIM* (codes for DNA cytosine-5-methyltransferase) and cg1018 encoding a putative ATP-dependent DNA helicase gene showed higher mRNA levels after exposure to ciprofloxacin. This included 8 of the 48 genes of the LexA regulon [40]. Further genes induced by ciprofloxacin were genes related to transcription, translation or protein modification or unknown functions. Among the genes showing reduced mRNA levels after exposure to ciprofloxacin was the *mraZ* gene, which putatively is involved in cell division.

**Table 1:** Differential gene expression of *C. glutamicum* caused by ciprofloxacin

Gene ID^a^	Gene name^a^	Function of protein^a^	M-value^b^	*P*-value^*^
DNA synthesis, repair, modification				
cg2141	*recA*	Recombinase A	3.86	0.000
cg1996	*cglIM*	DNA (cytosine-5-)-methyltransferase	3.41	0.000
cg0886	*-*	Putative ATP-dependent DNA helicase superfamily II	2.41	0.003
cg1401	*ligA*	DNA ligase (NAD(+))	2.16	0.002
cg1400	*-*	Putative DNA polymerase III, Gram-positive-type alpha subunit	2.01	0.002
cg1997	*cglIR*	Putative type II restriction endonuclease	1.38	0.001
cg0885	*-*	Putative helicase, UvrD/Rep-family	1.08	0.029
cg2509	*recO*	DNA repair protein RecO	1.05	0.044
cg1316	*-*	DNA/RNA helicase, SNF2 family	1.03	0.022
cg1018	*-*	Putative ATP-dependent DNA helicase	3.12	0.001
cg2950	*radA*	Putative ATP-dependent protease involved in DNA repair	1.10	0.029
Transcription, Translation, Proteinmodification				
cg2114	*lexA*	transcriptional regulator, LexA-family	1.61	0.004
cg3071	*pyrE*	Orotate phosphoribosyltransferase	1.38	0.025
cg0684	*papA*	Prolyl aminopeptidase A	1.31	0.006
cg0685	-	Conserved hypothetical protein similar to metal-dependent proteases, putative molecular chaperone	1.66	0.003
cg0686	-	Putative acetyltransferase, GNAT-family	1.32	0.013
cg1980	-	Hypothetical protein, MoxR-like ATPase	1.33	0.006
Genes of unknown function				
cg2113	*divS*	Cell division suppressor DivS	5.38	0.000
cg2381	-	Conserved hypothetical protein	3.86	0.000
cg1287	-	Conserved hypothetical protein	3.09	0.008
cg1962	-	Putative membrane protein	2.56	0.000
cg0839	-	Hypothetical protein	2.52	0.001
cg1977	-	Putative secreted protein	1.95	0.000
cg2026	-	Hypothetical protein	1.88	0.000
cg1978	-	Hypothetical protein	1.72	0.000
cg1917	-	Hypothetical protein	1.50	0.002
cg0841	-	Conserved hypothetical protein	1.39	0.016
cg1743	-	Conserved hypothetical protein	1.38	0.006
cg1937	-	Putative secreted protein	1.22	0.015
cg3018	-	Hypothetical protein	1.22	0.002
cg0451	-	Putative membrane protein	1.21	0.003
cg0712	-	Putative secreted protein	1.08	0.014
cg3106	-	Conserved hypothetical protein	1.03	0.029
cg2391	*aroG*	3-Deoxy-7-phosphoheptulonate synthase	-1.26	0.022
cg0203	*iolE*	Putative myo-inosose-2 dehydratase	-1.25	0.009
cg1342	*narJ*	Respiratory nitrate reductase 2, delta chain	-1.13	0.041
cg2378	*mraZ*	Putative MraZ protein	-1.13	0.023
cg2118	*fruR*	transcriptional regulator of fructose metabolism	-1.08	0.014
cg0205	*iolH*	Myo-inositol catabolism protein	-1.06	0.044
Genes of unknown function				
cg1918	-	Putative secreted protein	-2.82	0.000
cg2080	-	Conserved hypothetical protein	-1.74	0.012
cg2952	-	Putative secreted protein	-1.58	0.002
cg0045	-	ABC-type putative sugar transporter, permease subunit	-1.36	0.025
cg1884	-	Putative membrane protein	-1.30	0.038
cg1340	-	Conserved hypothetical protein	-1.27	0.001
cg3226	-	Putative MFS-type L-lactate permease	-1.15	0.009

^a^Gene ID, gene name and function of proteins are given according to CoryneRegNet (http://coryneregnet.de). ^b^Relative RNA levels of cells treated with 4 μg∙ml^-1^ ciprofloxacin compared to untreated cells are shown as log 2 values (M-values). ^*^ *P*-values were determined by Student’s *t*-test. Only genes with significant (*p* < 0.05) expression differences and M-values >1 or <1 are listed. The wild type was cultured in triplicate in CGXII with 1 % (w/v) glucose to an OD_600_ of about 5. Afterwards, cells were exposed to 4 µg/ml ciprofloxacin, a concentration allowing minor growth of the cultures. The data are available as Gene Expression Omnibus GSE77189 data set at http://www.ncbi.nlm.nih.gov/geo/

### Eliciting glutamate production by exposure to ciprofloxacin

**Fig. 2:**
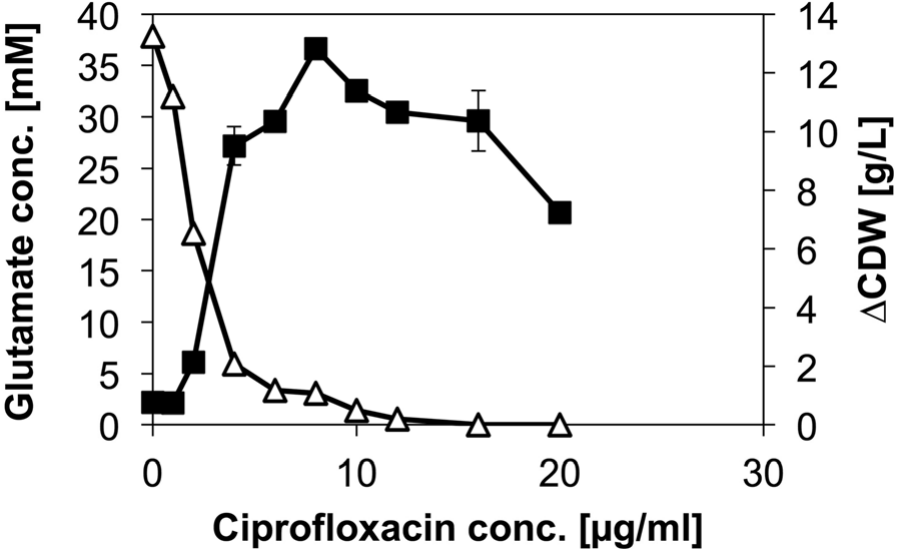
Biomass formation and glutamate production after ciprofloxacin addition. The *C. glutamicum* wild type, supplemented with 4 % (w/v) glucose, was grown to an optical density of 15 and ciprofloxacin in different concentrations was applied. After ciprofloxacin addition, cultures were incubated until glucose was consumed and the cell dry weight produced in this phase (∆CDW, open diamonds) and the glutamate concentration (black squares) were determined, after the consumption of the substrate. Values and error bars represent the mean and the experimental imprecision of duplicates

To investigate if a growth arrest due to ciprofloxacin maintains metabolically active *C. glutamicum* cells, culture supernatants of cells exposed to ciprofloxacin were assayed for amino acids. Interestingly, it was revealed that *C. glutamicum* produced glutamate when exposed to ciprofloxacin, even though other elicitors of glutamate production were absent from the medium (such as biotin limitation, Penicillin G, ethambutol, Tween 40 [11, 16, 19, 41]). Thus, although ciprofloxacin did not affect mRNA levels of genes of glutamate biosynthesis (Table 1), it elicted glutamate production. To identify the optimal ciprofloxacin concentration for triggering glutamate production by *C. glutamicum* WT, different concentrations of ciprofloxacin were added to cultures at an optical density of 15. It could be shown that even the addition of 2 μg/ml ciprofloxacin elicited the production of glutamate (Fig. 2). The highest glutamate titer was obtained by the addition of 8 μg/ml ciprofloxacin (37 ± 1 mM) which corresponded to a substrate specific glutamate yield of 0.13 g/g. This yield is comparable to glutamate production triggered for example by biotin limitation (0.15 g/g), addition of ethambutol (0.2 g/g) or penicillin G (0.25 g/g) [19, 33, 42]. Since the hitherto known triggers of glutamate production lead to reduced 2-oxoglutarate dehydrogenase complex (ODHC) activity [23, 24], it was analyzed whether the ODHC activity was decreased after exposure to ciprofloxacin. *C. glutamicum* was cultured in glucose minimal medium to an OD_600_ of about 8 before either no or 8 μg/L ciprofloxacin were added. ODHC activity was assayed in crude extracts prepared after 4 h of ciprofloxacin exposure. Indeed, when ciprofloxacin was added, ODHC activity was decreased by 87 % from 5.8 ± 0.7 mU/mg protein to 0.8 ± 0.3 mU/mg protein. These data are comparable to studies with Penicillin G or Tween 40 treated *C. glutamicum* cells, where the ODHC activities were decreased to a similar extent [22].

### Contribution of the mechanosensitive channel protein YggB

**Fig. 3:**
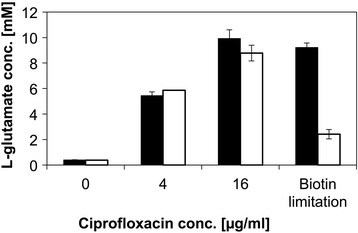
Difference of ciprofloxacin and biotin limitation after *yggB* deletion. The strains MB001 (black) and MB001∆*yggB* (white) were cultured to an optical density of 2 to 5 in CGXII supplemented with 1 % (w/v) glucose and ciprofloxacin was applied. In addition, the MB001 strain (black) was compared to MB001∆*yggB* (white) during biotin limitation. Therefore the pre-limited cells (in CGXII, 4 % glucose, 0 μg biotin per L) were re-inocculated to CGXII, containing 2 μg biotin per L, supplemented with 1 % (w/v) glucose. The glutamate concentration of both conditions was determined in the culture supernatant after the complete consumption of glucose. Values and error bars represent the mean and the standard error of triplicate cultivations

Besides reduced ODHC activities being involved in glutamate production, active glutamate export is a hallmark of glutamate production by *C. glutamicum*. The mechanosensitive channel protein MscS encoded by *yggB* (cg1432) is involved in the export of glutamate and in its absence glutamate production is reduced about four to five fold [26]. To test whether YggB is important for glutamate production triggered by ciprofloxacin addition, the gene was deleted and glutamate production of the respective strain was measured and compared to the parental strain (Fig. 3). The cells were grown in CGXII (1 % (w/v) glucose) and ciprofloxacin (0, 4, and 16 μg/L, respectively) was added at an optical density of 2 to 5. Unexpectedly, ciprofloxacin-induced production of glutamate was observed in the presence and absence of *yggB*. By contrast, glutamate production under biotin-limiting conditions was decreased about four fold, but not completely abolished (Fig. 3). Thus, unlike for glutamate production under biotin-limiting conditions, ciprofloxacin-triggered glutamate production was not affected by the absence of YggB.

### Influence of ciprofloxacin on ornithine, arginine, putrescine and lysine producing strains

**Fig. 4:**
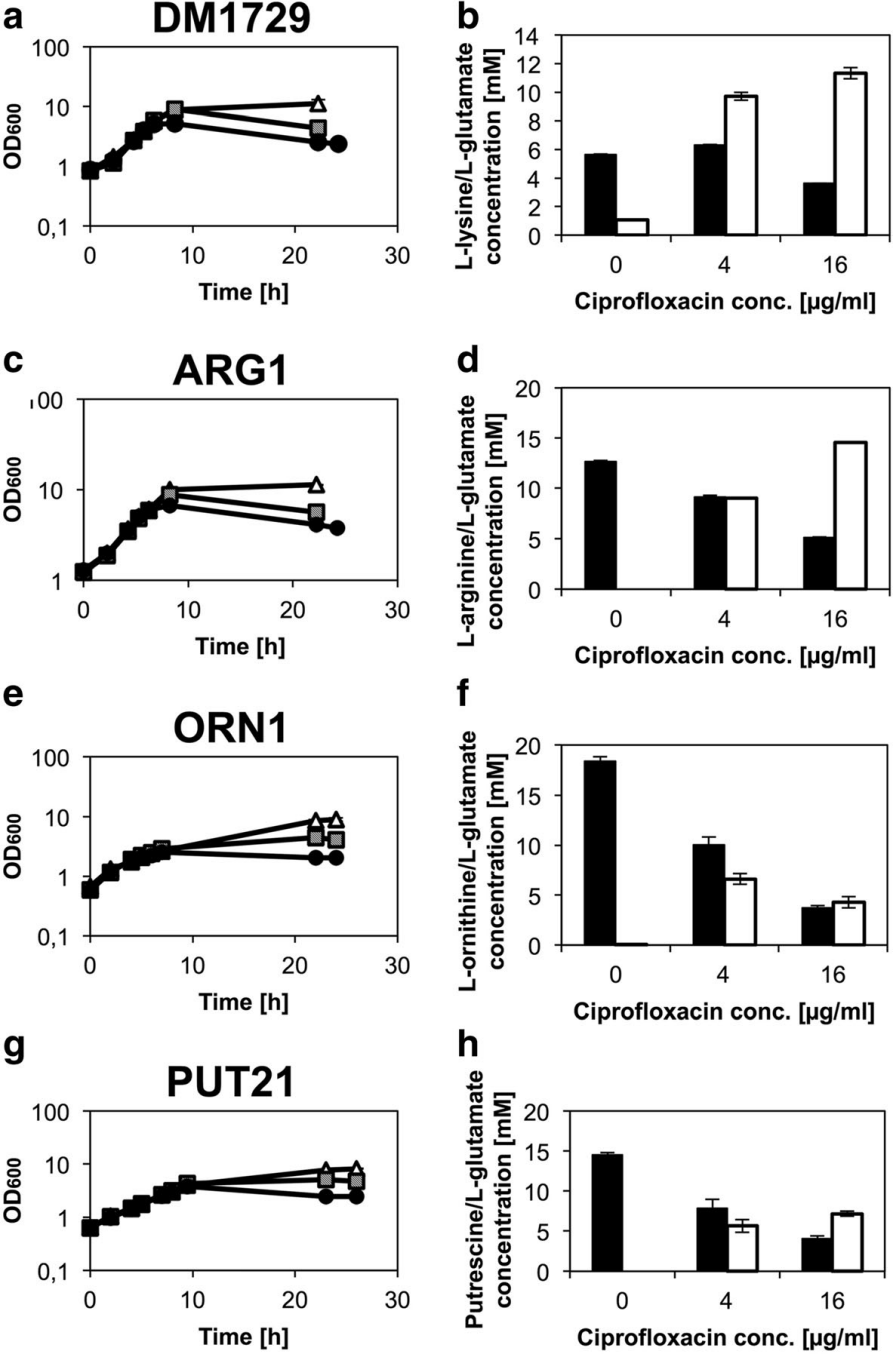
Growth of amino acid and diamine producer strains exposed to ciprofloxacin. The strains DM1729 (lysine producer) (**a** + **b**), ARG1 (arginine producer) (**c** + **d**), ORN1 (ornithine producer) (**e** + **f**) and PUT21 (putrescine producer) (**g** + **h**) were cultured to an optical density of 2 to 5 in CGXII supplemented with 1 % (w/v) glucose and ciprofloxacin was applied. Graphs on the left side (**a**, **c**, **e**, **g**) show the growth inhibition due to the addition of ciprofloxacin in concentrations of 0 μg/ml (white tirangles), 4 μg/ml (hatched squares) and 16 μg/ml (black circles). The graphs on the right side (**b**, **d**, **f**, **h**) show the concentrations of either lysine, arginine, ornithine or putrescine (black bars) and of glutamate (white bars) after the complete consumption of glucose. Values and error bars represent the mean and the standard error of duplicates

In order to test if ciprofloxacin addition triggers production of other glutamate-family amino acids, ornithine and arginine producing strains (ORN1 and ARG1) as well as strain PUT21 producing putrescine, a diamine derived from ornithine, were exposed to ciprofloxacin. The strains were cultured in CGXII supplemented with 1 % (w/v) glucose to an optical density of 2 to 5 before ciprofloxacin was added (0, 4, and 16 μg/L, respectively). However, the addition of ciprofloxacin reduced rather than increased production of ornithine, arginine and putrescine, respectively, and triggered the production of glutamate as by-product (Fig. 4). In a similar experiment, the effect of ciprofloxacin on lysine production by the lysine producing strain DM1729 was determined. Lysine production media have high biotin concentrations (a) to ensure sufficient levels of the biotin protein pyruvate carboxylase and (b) to avoid glutamate formation triggered by biotin limitation [43]. Addition of ciprofloxacin to the lysine producer resulted in glutamate production and lysine production was reduced at 16 μg/L ciprofloxacin (Fig. 4). Thus, ciprofloxacin addition specifically triggers glutamate production and interferes with production of glutamate-derived products (ornithine, arginine and putrescine) as well as with production of lysine, an amino acid not belonging to the glutamate-family of amino acids.

### Influence of ciprofloxacin on the production of overflow metabolites

Glutamate may be considered an overflow metabolite, which, however, requires sufficient supply of a nitrogen source. Efficient 2-oxoglutarate production requires deletion of the genes for enzymes converting 2-oxoglutarate to glutamate and nitrogen-limiting conditions [44]. To investigate whether ciprofloxacin triggers 2-oxoglutarate production under nitrogen-liming conditions, *C. glutamicum* WT was cultivated in CGXII medium containing ten times less nitrogen sources (2 g (NH_4_)_2_SO_4_ and 0.5 g/L urea) as compared to regular CGXII medium. Ciprofloxacin (16 μg/ml) was added to the culture, when growth with glucose stagnated due to nitrogen starvation and cultivation was continued until exhaustion of the carbon source. Ciprofloxacin did not affect biomass formation under these conditions, but the product spectrum was changed (Table 2). Instead of glutamate, 2-oxoglutarate was the main product formed (Table 2). Besides 18.6 ± 0.1 mM 2-oxoglutarate, 4 mM glutamate was formed when ciprofloxacin was added while formation of acetate and lactate was not increased by addition of ciprofloxacin (Table 2).

**Table 2:** Production of organic acids by *C. glutamicum* WT under nitrogen-limiting conditions in the absence or presence of ciprofloxacin

Ciprofloxacin [μg/ml]	Cell dry weight [mg/ml]	Acetate [mM]	Lactate [mM]	glutamate [mM]	2-oxoglutarate [mM]
0	6 ± 1	7 ± 2	7 ± 1	0 ± 1	7 ± 1
16	6 ± 1	10 ± 1	6 ± 1	4 ± 1	19 ± 1

Cells were cultivated in CGXII containing ten times less nitrogen sources than regular CGXII medium. 1 % glucose was used as carbon and energy source. At an OD_600_ of about 15, no or 16 μg/ml ciprofloxacin were added. The concentration of organic acids was determined after glucose depletion. All values represent the mean and the standard error of triplicates

## Discussion

Here, we have characterized how glutamate production by *C. glutamicum* can be triggered by addition of the gyrase inhibitor ciprofloxacin. In fact, it is known for long that gyrase inhibitors like novobiocin can elicit glutamate efflux in corynebacteria [37]. However, all triggers of glutamate production analyzed to date to some molecular detail have in common to affect the cell membrane and/or cell wall. For example, biotin limitation and addition of the fatty acid synthase inhibitor cerelunin impair fatty acid and/or mycolic acid biosynthesis, penicillin G targets peptidoglycan cross-linking, ethambutol inhibits cell wall arabinogalactan biosynthesis, detergents like Tween 40 impair the surface integrity. The mechanism of ciprofloxacin action with respect to triggering glutamate production remains to be elucidated. In the simplest case, growth arrest by ciprofloxacin maintains metabolic activity of the cells which convert growth substrates to glutamate as overflow metabolite. This notion is supported by the fact that under nitrogen-limiting conditions 2-oxoglutarate, the immediate nitrogen-free precursor of glutamate is produced instead of glutamate (Table 2).

**Fig. 5:**
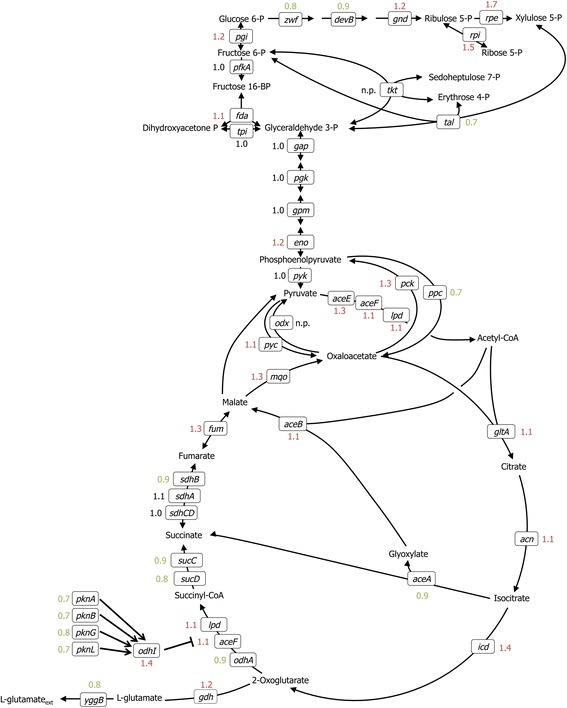
Scheme of the central carbon metabolism and glutamate biosynthesis and relative RNA levels with/without ciprofloxacin treatment. Genes are depicted next to the reaction catalyzed by the encoded enzymes. Relative RNA levels of cells treated with 4 µg/ml ciprofloxacin compared to untreated cells are shown (values in green are below 1, those in red greater than 1), however, unlike the genes listed in Table 2, none of the genes depicted here showed significantly changed expression as determined by Student’s *t*-test, i.e., *p* > 0.05. 6PGL: 6-phosphogluconolactone; 6PG: 6-phosphogluconat

The exposure of *C. glutamicum* to ciprofloxacin altered expression of remarkably few genes (Table 1). Genes of glutamate biosynthesis were not significantly altered (Fig. 5). By contrast, one study reported decreased expression of almost all genes involved in the EMP pathway, the PPP, and the TCA cycle by cells triggered for glutamate by addition of detergent, penicillin or by biotin limitation for 12 hs [45]. These expression changes have since been observed when gene expression is compared between slow and fast growing cells [46]. To minimize secondary effects due to long exposures, cells treated with ciprofloxacin were analysed already 1 h after addition of ciprofloxacin (Table 1). Accordingly, a coherent picture of differential gene expression emerged. The primary transcriptional response to ciprofloxacin targets DNA synthesis as in *Streptomyces coelicolor* [47]. The response of *C. glutamicum* to ciprofloxacin showed the typical expression pattern of the SOS response as consequence of DNA damage conditions [40]. Namely the genes *recA*, *cglM*, *cglR*, *radA*, *lexA* (autoregulation) and *divS* and several genes of unknown function (cg2381, cg2026, cg0841, cg1977) of the 48 genes of the LexA regulon were upregulated. As known for *C. glutamicum* and other bacteria, autoproteolytic cleavage of the transcriptional regulator LexA is induced by RecA bound to single stranded DNA leading to the induction of the LexA regulon [48–51]. Several genes of the LexA regulon [40] were induced by ciprofloxacin in *C. glutamicum*. Notably, the LexA regulon was induced in *E. coli* by nalidixic acid, which is a gyrase inhibitor of the class of quinoles as ciprofloxacin [52]. Transcriptional regulation by LexA is not a prerequisite of glutamate production in *C. glutamicum* since induction of the LexA regulon has not been reported when glutamate production was triggered by biotin limitation or by addition of ethambutol or tween 40 [45, 53]. When *C. glutamicum* was treated with high ciprofloxacin concentrations, very few colony forming units were observed and these showed colony heterogeneity as depicted in Additional file 1: Figure S1 for 100 μg/ml ciprofloxacin, a concentration about 60 fold higher than IC_50_. Likely, mutations have occurred leading to different ciprofloxacin susceptibility as is often observed for treatments triggering the LexA regulon and the SOS response [54].

Genome resequencing of glutamine-producing *E. coli* mutants obtained by classical mutagenesis and screening revealed nonsynonymous mutations in *gyrA* which encodes the primary target of ciprofloxacin DNA gyrase and these mutations were shown to have caused glutamine overproduction and reduction of chromosomal DNA supercoils [55]. Similarly, overexpression of genes encoding topoisomerase I (*topA*) and topoisomerase IV (*parC* and *parE*) reduced chromosomal DNA coils and entailed glutamine production by *E. coli* [55]. MurI-type glutamate racemases are known to inhibit DNA gyrase activity in *E. coli* [56] and *Bacillus subtilis* [57]. These enzymes link DNA gyrase activity to murein biosynthesis since D-glutamate is present in peptidoglycan cross-links. As the closely related *C. diphtieriae* possesses D-glutamate in its tetrapeptides (L-Ala-D-Glu-meso-Dap-D-Ala) and tripeptides (L-Ala-D-Glu-meso-Dap) of peptidoglycan [58], it is likely that D-glutamate is also present in the peptidoglycan peptides of *C. glutamicum*. In fact, *C. glutamicum* possesses a *murI* gene [59]. However, excretion of D-amino acids by *C. glutamicum* has not been observed unless a heterologous racemase gene was overexpressed [60]. Altered murein biosynthesis and cell wall integrity due to ciprofloxacin may be involved in triggering glutamate production under these conditions.

Exposure to ciprofloxacin triggered glutamate production even in strains overproducing other amino acids such as lysine as it is true for penicillin G-triggered glutamate production [16]. Thus, ciprofloxacin is a specific trigger of glutamate production by *C. glutamicum*. Glutamate was also produced efficiently in the absence of *yggB* coding for the glutamate channel, which releases glutamate by passive diffusion [61]. By contrast, the deletion of *yggB* reduced, but not completely abolished glutamate production triggered by biotin limitation (see also Fig. 3) or Penicillin G [26, 62]. The residual glutamate production in the absence of YggB varied from trigger to trigger, but suggested that (an) additional glutamate export system(s) may exist. This is supported by the fact, that the export of glutamate is also observed when the external concentration exceeds the intracellular concentration, which suggests an additional energy-dependent transport mechanism [63].

Triggers of glutamate production are known to elicit a metabolic switch in the sense that ODHC activity is reduced [23, 24], and also ciprofloxacin reduced ODHC activity about seven fold (see above). The reduced ODHC activity in the presence of ciprofloxacin may also explain ciprofloxacin-triggered production of 2-oxoglutarate under nitrogen-limiting conditions (Table 2). Other triggers of glutamate production also led to 2-oxoglutarate production under nitrogen-limiting conditions [44, 64, 65]. When *aceA* (encoding isocitrate lyase), *gltB* (encoding glutamate-2-oxoglutarate aminotransferase) and *gdh* (encoding glutamatate dehydrogenase) were disrupted in addition, 2-oxoglutarate production was improved 16 fold and almost 50 g/L 2-oxoglutarate accumulated [65].

## Conclusions

Glutamate production by *C. glutamicum* triggered by ciprofloxacin was characterized and shown not to be affected by the absence of the putative glutamate export system YggB. This gyrase inhibitor led to increased expression of genes that are involved in DNA synthesis, repair and modification and belong to the LexA regulon and SOS response of *C. glutamicum*. The exact mechanism(s) of triggering glutamate production by ciprofloxacin and other previously published triggers in *C. glutamicum* remain(s) enigmatic. However, as observed with all published triggers of glutamate production, ciprofloxacin reduced ODHC activity in *C. glutamicum*. Moreover, production of 2-oxoglutarate could be triggered by ciprofloxacin under nitrogen-limiting conditions.

## Methods

### Microorganisms and growth conditions

Microorganisms and plasmids used in this study are listed in Table 3. *E. coli* DH5α was used for gene cloning. *C. glutamicum* and *E. coli* strains were routinely grown in lysogeny broth (LB) (10 g/L tryptone, 5 g/L yeast extract, 10 g/L sodium chloride) in 500 mL baffled flasks on a rotary shaker (120 rpm) or LB agar plates (18g/L agar) at 30 °C or 37 °C. For growth experiments, CGXII minimal medium [66] was used for *C. glutamicum*. Growth was followed by measuring the optical density at 600 nm using a V-1200 Spectrophotometer (VWR, Radnor, PA, USA). An OD_600_ of 1 corresponds approximately to an estimated cell dry weight of 0.25 g/L.

**Table 3:** Strains and plasmids used in this study

*E. coli* strains		
DH5α	F^−^ *thi*-1 *endA*1 *hsdr*17(r^−^, m^−^) *supE*44 ∆*lacU*169 (Φ80*lacZ*∆M15) *recA*1 *gyrA*96 *relA*1	[74]
*C. glutamicum* strains		
WT	Wild type strain ATCC13032, auxotrophic for biotin	ATCC
MB001	ATCC 13032 with in-frame deletion of prophages CGP1 (cg1507-cg1524), CGP2 (cg1746-cg1752), and CGP3 (cg1890-cg2071)	[75]
ARG1	WT with in-frame deletion of ∆*argR* carrying the pEKEx-*argB* ^fbr^ vector	[76]
ORN1	WT with in-frame deletion of ∆*argFR*	[76]
DM1729	WT with *lysC* ^P458S^, *hom* ^V59A^, *pyc* ^T311I^	[70]
PUT21	WT with in-frame deletion of ∆*argFR* carrying the pVWEx1-*speC*-*argF*_*leaky*_ vector	[77]
MB001∆*yggB*	MB001 with in-frame deletion of ∆*yggB*	This study
Plasmids		
pK19*mobsacB*	Kan^r^, mobilizable *E. coli* vector for the construction of insertion and deletion mutants of *C. glutamicum* (oriV, sacB, lacZ)	[78]
pK19∆*yggB*	Kan^R^, pk19mobsacB with the deletion construct of gene *yggB*	This study

When necessary, the growth medium was supplemented with kanamycin (25 μg/mL), spectinomycin (100 μg/mL), isopropyl β-D-1-thiogalactopyranoside (IPTG) (1 mM) and arginine (750 μM). The growth behavior, amino acid and organic acid production and the substrate consumption of *C. glutamicum* strains were analyzed in 500 ml baffled flasks. Briefly, a 50 mL BHI (37 g/L) seed culture was inoculated from an agar plate and cultivated overnight. The cells were harvested by centrifugation (4,000 x g, 10 min) and washed twice with CGXII minimal medium without carbon source. Subsequently, 50 mL CGXII medium, containing a given concentration of carbon source and necessary supplements, was inoculated to an optical density of 1.0. Detailed information on the carbon source and nitrogen concentrations employed is given in the results chapter.

### Determination of ODHC activity

Cultivation of the *C. glutamicum* wild type was performed in CGXII (4 % glucose) and ciprofloxacin was added at an OD_600_ of 10. After 4 °C, the cells were harvested and immediately, crude extracts were isolated by ultrasonic treatment and the fresh extracts were analyzed as described before [67].

### Molecular genetic techniques

Standard methods such as restriction digestions, and ligation were carried out as described elsewhere [68]. Digested DNA was purified by using the QIAquick Gel Extraction Kit (Qiagen, Hilden, Germany). *E. coli* cells were transformed by heat shock [68] and *C. glutamicum* cells were transformed by electroporation [66]. Isolation of genomic DNA was performed as previously described [69]. Chromosomal changes in *C. glutamicum* were performed as described elsewhere [66]. The gene for the putative glutamate exporter was deleted in MB001 pK19mob*sacB*∆*yggB*. Flanks of yggB were amplified and joined by crossover-PCR with primers yggB_up_fw + yggB_up_rw and yggB_dw_fw + yggB_dw_rv (italics: restriction sites, underlined: homologous sequence; yggB_up_fw, CTT*GAATTC*GGACCCGTCCAAGCCAAG (*Eco*RI); yggB_up_rw, AGAGACGACCTAAGCCAGTCTGGGTACGCCTAAAATCATGAGC; yggB_dw_fw, AGACTGGCTTAGGTCGTCTCTGTCCAAGAGACAGTTGCGCC; yggB_dw_rv, CCT*CTGCAG*GGAAGGGAGTTGAAGGTGACG (*Pst*I)). The crossover PCR prodcut was restricted with *Eco*RI and *Pst*I and ligated into *Eco*RI and *Pst*I restricted pK19*mobSacB*. The primers yggB_up (CTTTTGGCGCTCCAAGTACT) and yggB_down (TCCTCGAGCGATCGAACAAT) were used for confirmation of the by PCR amplification and DNA sequencing.

### Determination of amino acid and carbohydrate concentrations

For the quantification of extracellular amino acids and carbohydrates, a high-performance liquid chromatography system was used (1200 series, Agilent Technologies Deutschland GmbH, Böblingen, Germany). Samples were withdrawn from the cultures, centrifuged (13,000 x g, 10 min), and the supernatant used for analysis.

Organic acids were analyzed on a normal phase column (organic acid resin 300 x 8 mm, 10 μm particle size, 25 Å pore diameter; Chromatographie Service GmbH, Langerwehe, Germany) using 5 mM sulfuric acid as the mobile phase at a flow rate of 1 mL min^-1^ and were detected with a refractive index detector (RID G1362A, 1200 series, Agilent Technologies). Amino acids were automatically modified by precolumn derivatisation with ortho-phthalaldehyde and separated as described previously [70]. ornithine, lysine and glutamate were quantified using a pre-column (LiChrospher 100 RP18 EC-5 μ (40 x 4 mm), CS-Chromatographie Service GmbH, Langerwehe, Germany) and a reversed phase column (LiChrospher 100 RP18 EC-5 μ (125 x 4 mm), CS Chromatographie) as a main column and detected with a fluorescence detector at excitation at 230 nm and 450 nm emission (FLD G1321A, 1200 series, Agilent Technologies). For the determination of arginine and putrescine, a reverse-phase (RP) LiChrospher 100 RP8 EC-5 μ precolumn (40 x 4.6 mm) and a RP8 EC-5 μ (125 x 4.6 mm) main column (CS Chromatographie, Langerwehe, Germany) were used. 100 μM L-asparagine was used as an internal standard. The mobile phases used were in case of RP8 A: 0.25 % Na-acetate pH 6, B: methanol. The gradient used was: 0 min 30 % B, 1 min 30 % B, 6 min, 70 % B, 11 min 90 % B, 14 min 70 % B, 16 min 30 % B. In case of RP18, the mobile phases used were A:0.1 M Na-acetate pH 7.2, B: methanol. The gradient used was: 0 min 20 % B, 0.5 min 38 % B, 2.5 min 46 % B, 3.7 min 65 % B, 5.5 min 70 % B, 6 min 75 % B, 6.2 min 85 % B, 6.7 min 20 % B.

### Transcriptome analysis using DNA microarrays

The *C. glutamicum* wild type was exposed to 4 μg/ml ciprofloxacin to enable growth of the cells and compared to the untreated wild type. The cells were inoculated in CGXII (4 % (w/v) glucose), ciprofloxacin was added at an OD_600_ of 5 and the RNA was isolated after one hour of ciprofloxacin exposure. Fluorescently labeled cDNA synthesis and DNA microarray hybridization was performed as described previously [71, 72]. The data was analyzed as described previously [73]. The data were normalized using the LOWESS approach. The significance of gene expression rates was determined using a *t*-test adjusted with the False Discovery Rate approach. Furthermore, the adjusted *p*-value had to be lower than 0.05 and the genes needed to be regulated more than two-fold. The data are available as Gene Expression Omnibus GSE77189 data set at http://www.ncbi.nlm.nih.gov/geo/.

## Additional file

Additional file 1: Figure S1. Colony formation of *C. glutamicum* wild type without ciprofloxacin (0 μg/ml) and with the addition of 100 μg/ml to the medium. (DOCX 968 kb)

## Abbreviations

cfu: colony forming units
Msc: Mechanosensitive channel protein
ODHC: 2-oxoglutarate dehydrogenase complex
