# Supplementary material for: Ciprofloxacin triggered glutamate production by Corynebacterium glutamicum
Source: BMC Microbiol. 2016 Oct 7;16:235. doi: 10.1186/s12866-016-0857-6 (PMC5055667; doi:10.1186/s12866-016-0857-6)
Supplement: Additional file 1: Figure S1. — Colony formation of C. glutamicum wild type without ciprofloxacin (0 μg/ml) and with the addition of 100 μg/ml to the medium. (DOCX 968 kb) [file 12866_2016_857_MOESM1_ESM.docx]

**Supporting data**

**Figure S1:** Colony formation of *C. glutamicum* wild type without ciprofloxacin (0 µg/ml) and with the addition of 100 µg/ml to the medium.
